# Performance Analysis of an α-Graphyne Nano-Field Effect Transistor

**DOI:** 10.3390/mi14071385

**Published:** 2023-07-06

**Authors:** Habibullah Khan, Md. Monirul Islam, Rajnin Imran Roya, Sariha Noor Azad, Mahbub Alam

**Affiliations:** Department of Electrical and Electronic Engineering, Bangladesh University of Engineering and Technology, Dhaka 1000, Bangladesh; 1706040@eee.buet.ac.bd (H.K.); 1706042@eee.buet.ac.bd (M.M.I.); 1706088@eee.buet.ac.bd (R.I.R.); 1706051@eee.buet.ac.bd (S.N.A.)

**Keywords:** graphyne nanoribbon, α-graphyne nanoribbon, ballistic field effect transistor (FET), tight binding model, non-equilibrium Green’s function (NEGF), single vacancy defect, edge defect

## Abstract

Graphyne has attractive electronic properties that make it a possible replacement of silicon in FET technology. In FET technology, the goal is to achieve low power dissipation and lower subthreshold swing. In this study, we focused on achieving these goals and studied the electronic properties of α-graphyne nanoribbons. We simulated the transfer and output characteristics of an α-graphyne ballistic nanoribbon FET. We used the tight-binding model with nearest-neighbor approximation to obtain the band structure which gives the same band structure as the one found from the DFT. In order to simulate the I-V characteristics of the transistor we used the non-equilibrium Green’s function (NEGF) formalism. The results show that the modeled FET can provide a high Ion/Ioff ratio and low subthreshold swing. We also studied the effects of defects as defects cannot be avoided in any practical device. The study shows that the Ion/Ioff ratio and subthreshold swing improves as defects are added, but the delay time and dynamic power dissipation worsen.

## 1. Introduction

Moore’s Law, first observed in 1965, predicts that the number of transistors on a microchip doubles approximately every two years, which has led to exponential growth in computing power. This trend has enabled a wide range of technological advancements and has transformed the way we live and work. However, the shrinking of transistor size is pushing silicon-based technology towards its physical limits. As the transistor size decreases and the chip density increases, the leakage current becomes more prominent, quantum tunneling challenges reliable operation, it becomes difficult to accommodate the interconnections, and heat dissipation becomes more difficult [1]. These issues are slowing the progress of Moore’s law and increasing the costs and complexity of new device designs. This has led to a search for alternative materials that can provide better performance and scalability.

Carbon is the fundamental building block for most organic and biological molecules. It can have three distinct covalent bonds, namely, sp, sp_2_, and sp_3_ hybridized bonds, which contribute to its unique physical and chemical properties. The extended networks of sp3 and sp2 bonds give rise to several allotropes of carbon, i.e., diamond, graphite, fullerenes, graphene, carbon nanotubes, etc. Graphene is a two-dimensional (2D) hexagonal sheet of carbon atoms, which emerged as a potential replacement of silicon in FET technology because of its remarkable properties. In the bulk form, graphene has a zero bandgap. The band structure of graphene is, in fact, uniquely different from semiconductors, conductors, and insulators. It has a conical band structure (Dirac cone) above and below the Fermi level [2].

Graphyne is another allotrope of carbon that contains alternating single and triple bonds. It is a monolayer carbon lattice with different structural orientations. Graphyne contains alternating single and triple bonds [3]. It is sometimes referred to as “extended graphene” due to its structural similarity with graphene [4] but with additional acetylenic linkages between carbon atoms. The lattice structure of graphyne is similar to the graphene honeycomb structure, but the C-C bonds are periodically substituted by -C=C- bonds. Because such replacements can be formed at different C–C bonds, graphynes form multiple lattice structures. Based on the different combinations of –C≡C–, the structures of graphyne are classified into four categories [5], i.e., α-, β-, γ-, and (6,6,12)-graphyne. Though it has good electronics and transport properties, it has still received secondary importance among various forms of the carbon family including nanotubes and graphene. Importantly, graphyne’s structure is stable at room temperature, and it shows many special characteristics. Notable features of Graphyne are high thermal conductivity and high charge carrier mobility which can lead to promising applications in electronics and photonics [6]. In 1987, Baughman et al. first presented the theoretical idea of graphyne. However, the first successful methodology for synthesizing graphyne was developed in 2010 by Li et al. In 2019, another method was developed by Cui and co-workers using a mechanochemical technique for synthesizing graphyne using benzene and calcium carbide. The first scalable synthesis of γ-graphyne was successful in 2022 using the polymerization of 1,3,5-tribromo-2,4,6-triethynylbenzene [7]. The α-graphyne nanoribbon has a hexagonal lattice structure similar to graphene but with alternating single and triple bonds. Bulk graphyne is not a semiconductor. To make it semiconductive, which is crucial for transistors, it must be produced in the form of nanoribbons with the right dimensional ratios. Depending on the cutting edge, alpha-graphyne nanoribbons (α-GyNRs) can be of different typesm and each one has unique properties. In studies to date, not much attention has been paid to the transport properties and current voltage characteristics of graphyne nanoribbons, and there has been no previous study on graphyne nanoribbon field effect transistors (GyNR-FETs). In the case of transistor design, material choice is an integral part upon which the device’s performance is highly dependent. Material choice also depends on specific applications’ requirements and trade-offs between different properties. Because of it having high electron mobility and conductivity, graphyne has become a promising material for designing transistors. Here, high electron mobility indicates faster switching speeds and lower power consumption. Graphyne provides higher mobility and lower resistance in comparison with conventional materials, and that is the reason it can be used as a channel material in the FET’s channel region which actually controls the flow of the current between the source and drain terminals. Though transistor modeling with graphyne is at an early stage of research, it has the potential for developing high-performance and highly efficient electronic devices. In this study, we present a band structure and carrier density graphically corresponding to alpha-graphyne. Then, after showing that it is usable for transistor modeling, we demonstrate an alpha-graphyne-based field effect transistor and its transfer characteristics and performance parameters with and without single vacancy (SV) defects. Various device performance parameters such as Ion/Ioff ratio, subthreshold swing, and delay time are calculated, and the effect of SV defects on these parameters is shown.

## 2. Materials and Methods

**Figure 1:**
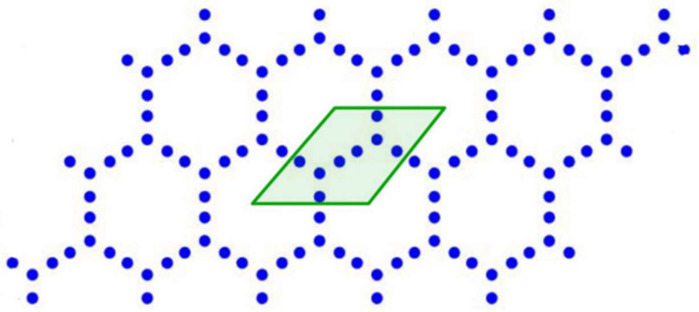
Geometry of α-graphyne.

First, we constructed the geometry of the transistor using alpha-graphyne [5]. The geometry of α graphyne is shown below in Figure 1. To do this, we fixed an atom at the center of the x-y plane. We call it the reference atom. Then, the position of the rest of the atoms in a unit cell was calculated using the trigonometric method.

**Figure 2:**
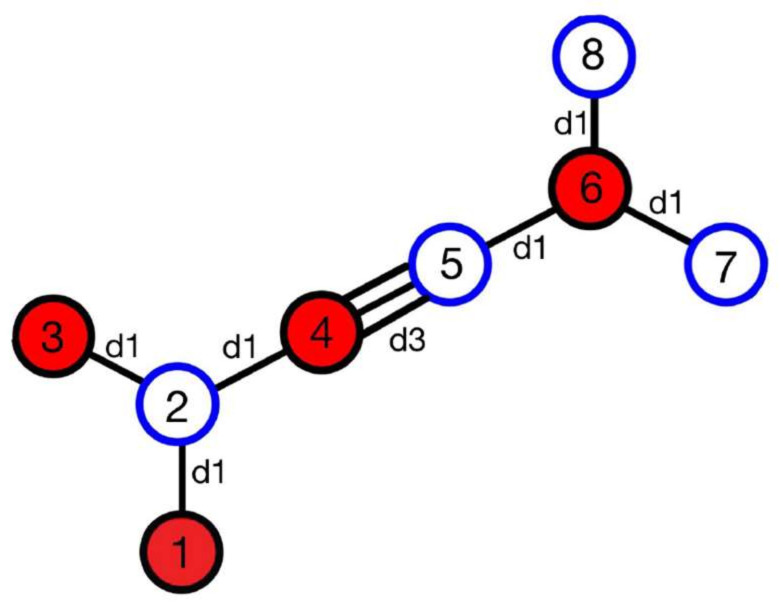
Unit cell of α-graphyne.

The lattice constant is a0 = 6.966 Å. The unit cell is shown in Figure 2.

We constructed our device by cutting a 2D atomic alpha-graphyne lattice at an angle θ = 60 degree.

d1 = 1.396 Å

d3 = 1.230 Å

The positions of each atom in the unit cell according to the numbering:

$$\begin{array}{cc} \left(x_{1,}y_{1}\right)=(0,0) & \\ \left(x_{2,}y_{2}\right)=(0,d1) & \\ \left(x_{3,}y_{3}\right)=(-d1*\sin,d1+d1*\cos\theta ) & \\ \left(x_{4,}y_{4}\right)=(d1*\sin\theta ,d1+d1*\cos\theta ) & \\ \left(x_{5,}y_{5}\right)=((d1+d3)*\sin\theta ,d1+(d1+d3)*\cos\theta ) & \\ \left(x_{6,}y_{6}\right)=((2d1+d3)*\sin\theta ,d1+(2d1+d3)*\cos\theta ) & \\ \left(x_{7},y_{7}\right)=((3d1+d3)*\sin\theta ,\;d1+(d1+d3)*\cos\theta ) & \\ \left(x_{8,}y_{8}\right)=((2d1+d3)*\sin\theta ,2d1+(2d1+d3)*\cos\theta ) & \end{array}$$

Then, we shifted this unit cell along the vertical axis by the along width: $2d1+d3+\left(2d1+d3\right)*\cos\theta$ and along length: (2d1 + d3) ∗ *sinθ* to form the width of the device.

After that, we shifted this width along the horizontal axis by $2\left(2d1+d3\right)*\sin\theta$ to form the length of the device.

**Figure 3:**
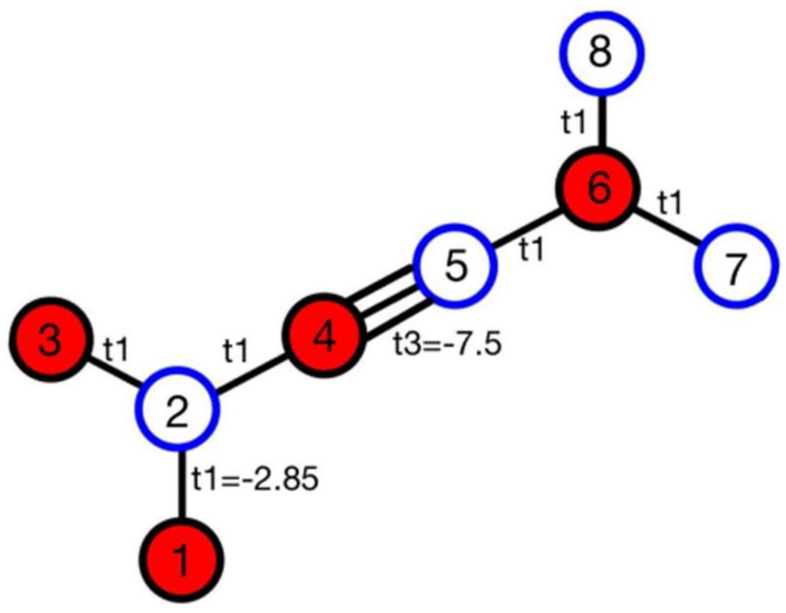
The couplings between the atoms in a unit cell.

Then, the Bloch Hamiltonian was formed using the device’s geometry and taking into account the coupling between the atoms [5]. The coupling between the atoms in a unit cell is shown in Figure 3.

We constructed the band structure using the tight binding model with nearest-neighbor approximation.

Matlab software was used to simulate the graphyne nano-FET. The band structure, the device’ density of states (DOS), current–voltage characteristics, and total capacitance curves were calculated using Matlab software. We used the tight-binding parameters that were extracted in a way that the band structure from the TB model resembled the band structure found from yjr DFT [5].

The simulation of the device is based on the non-equilibrium Green’s function (NEGF) formalism [8,9].

The NEGF theory was formulated by Keldysh in order to reproduce the solution of the Schrodinger equation [10]. It determines the carrier distribution of open quantum devices by consistently calculating the energy and occupancy of its scattering states.

The Green’s function gives the impulse response of the device. We defined the device Hamiltonian H, which is the energy information of the device, and the self-energies of the interactions, i.e., Σ1 (left lead) and Σ2 (right lead). The calculations are carried out in the energy domain and position basis, and the retarded Green’s function can be found from the equation [8,11,12]

(1) $$\left[\mathrm{EI}-H-\Sigma_{1}-\Sigma_{2}\right]G^{R}=I$$

Here, E is the energy of the electrons introduced into the device through the lead and I is the identity matrix. The modeling of the device Hamiltonian takes into account the nearest-neighbor approximation.

The potential profile throughout the device is solved self consistently [12]. The electron correlation function is given by [8,10,11,12]:

(2) $$G^{n}=G^{R}\Sigma^{\mathrm{in}}G^{A}$$

G^A^ is the advanced Green’s function given by $G^{A}={\left[G^{R}\right]}^{†}$. The scattering function Σ^in^ describes the rate at which electrons are scattered in the leads at a certain energy level.

$G^{n}$ represents a matrix version of the electron density, and we can plot the local density of states from the diagonal values of this matrix.

The equations for calculating this are [8,10,11,12,13,14,15]:

(3) $$\Sigma^{\mathrm{in}\;}=f_{1}\Gamma_{1}+f_{2}\Gamma_{2}$$

(4) $$\Gamma_{1}=i\left[\Sigma_{1}-{\Sigma_{1}}^{†}\right]$$

(5) $$\Gamma_{2}=i\left[\Sigma_{2}-{\Sigma_{2}}^{†}\right]$$

Γ_1_ and Γ_2_ are the broadening matrices for the source and drain contacts.

f_1_ and f_2_ are the Fermi–Dirac distribution functions.

The transmittance of the electron through the device is calculated using the equation [8,11,12,14,15]:

(6) $$T=\mathrm{Trace}\left[\Gamma_{1}G^{R}\Gamma_{2}G^{A}\right]$$

In order to calculate the current, we used the Landauer–Buttiker formula [11]

(7) $$I=\frac{2q}{h}\int_{-\infty }^{+\infty }T\left(E\right)\left\{f_{1}\left(E\right)-f_{2}\left(E\right)\right\}\mathrm{dE}$$

In order to calculate the total capacitance, we considered the gate capacitance and the fringing capacitance. The gate capacitance contains the oxide capacitance and the quantum capacitance. The oxide capacitance is a function of the device dimensions and can be approximated by [16] $C_{\mathrm{ox}}=\frac{\varepsilon_{0}\varepsilon_{r}W}{d}$ (per unit length of the channel). We used SiO_2_ as the device dielectric so the relative permittivity, ε_r_, is 3.9. W and d are the gate width (nanoribbon width) and oxide thickness, respectively.

For the metal–insulator–metal capacitor, the potential difference is the only relevant contribution. Therefore, we can calculate the capacitance using Gauss’ law. However, when semiconductive material is used as the capacitive plate, the capacitance cannot be calculated directly from the potential difference between the plates. When the potential is applied, the electron concentration in the negative plates increases and occupies higher energy states in the band structure, while the positive plate loses electrons, which creates an empty band of a low-energy state in the band structure. Therefore, the capacitance cannot be calculated only from device’s geometry and Gauss’ law. The effect of band filling and band emptying must be taken into account. This effect affects the total capacitance by imitating a second capacitance in series which is the quantum capacitance.

The quantum capacitance is obtained from [8] $C_{Q}=\frac{\partial \mathrm{Qc}}{\partial \mathrm{Vc}}$.

$Q_{C}$ is the channel charge and $V_{C}$ is the channel voltage. The gate capacitance is the equivalent of a series combination of the oxide and quantum capacitances.

(8) $$C_{g}^{-1}={C_{\mathrm{ox}}}^{-1}+{C_{Q}}^{-1}$$

According to the IRTS standard, the fringing capacitance is $C_{f}=2C_{g}$.

The total capacitance is a parallel combination of the gate capacitance and the fringing capacitance, i.e.:

(9) $$C_{\mathrm{tot}}=C_{g}+C_{f}.$$

The delay time can be calculated using the equation:

(10) $$\tau_{d}=\frac{C_{\mathrm{tot}}V_{b}}{I_{\mathrm{on}}}$$

where V_b_ is the bias voltage and I_on_ is the on current. The dynamic power dissipation is calculated as:

(11) $$\mathrm{DPD}=V_{b}I_{\mathrm{on}}\tau_{d}$$

## 3. Results and Discussion

The device we have simulated has a width of 1.69 nm and a channel length of 7.56 nm.

**Figure 4:**
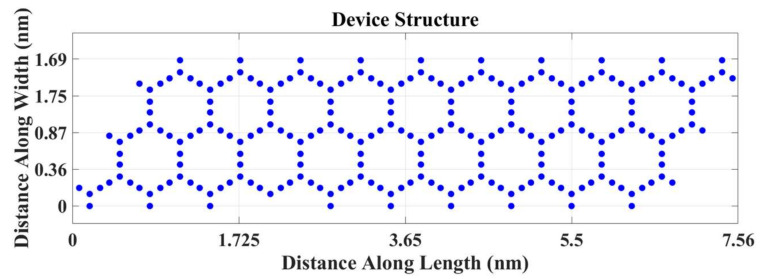
Structure of the channel.

The final device geometry is shown in Figure 4:

### 3.1. Band Structure

**Figure 5:**
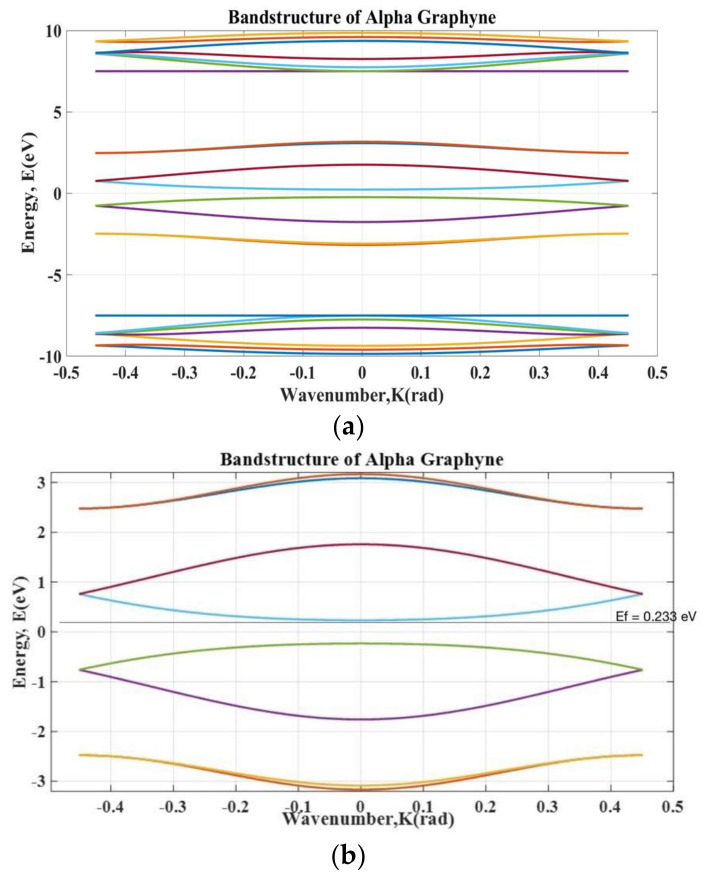
Band structure of the α-graphyne nanoribbon: (**a**) complete band structure and (**b**) band structure zoomed in at the operating region.

The electronic band structure gives information on the allowed quantum states of electrons. We plotted the band structure from the Bloch Hamiltonian matrix. The eigenvalues of the Bloch Hamiltonian give the energy matrix. Each column of the energy matrix represents energy for the allowed states. We demonstrate the band structure for the α- graphyne nanoribbon in Figure 5.

From the band structure, we can see that there is a bandgap of 0.46 eV. We can tune the bandgap by changing the device’s width. At this bandgap, we have fairly semiconducting behavior. Most importantly, the semiconducting behavior of the material is expected to control its conductivity. Actually, for this particular dimension (a width of 1.69 nm), the semiconducting behavior of the band structure allows one to control the conductivity in the particular region. The bands in this region give stable switching operation.

### 3.2. Local Density of States (LDOS)

**Figure 6:**
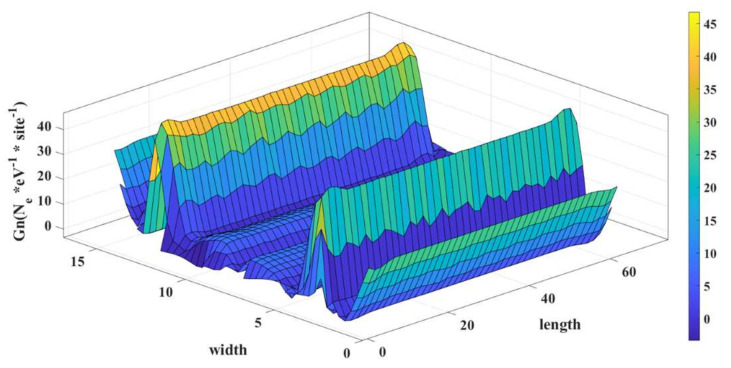
LDOS of the α-graphyne nanoribbon in ON condition at E_F_ = 0.233 eV.

We calculated the local density of states from the diagonal elements of the electron correlation function, $G^{n}$, as described in Section 2. The diagonal elements of the electron correlation matrix (also known as te density matrix), $G^{n},$ give the electron density profile throughout the device. We plotted the density with respect to the length and width of the device, and we can see the solutions of the Schrodinger equation in terms of electron density. This is shown in Figure 6. Changing the energy level for electron insertion translates to moving the Fermi level to a different energy, and the electron density profile will be different.

From Figure 6, we can see the local density of state for the alpha-graphyne for the Fermi energy of 0.233 eV, which corresponds to the on state of the device. The unit of LDOS is the number of electrons per eV per atomic site. N_e_ represents the number of electrons.

In this state, we can see that there is a significant electron concentration along the length of the device. Therefore, if an external voltage is applied across the length, then there will be a considerable amount of current conduction along the length.

**Figure 7:**
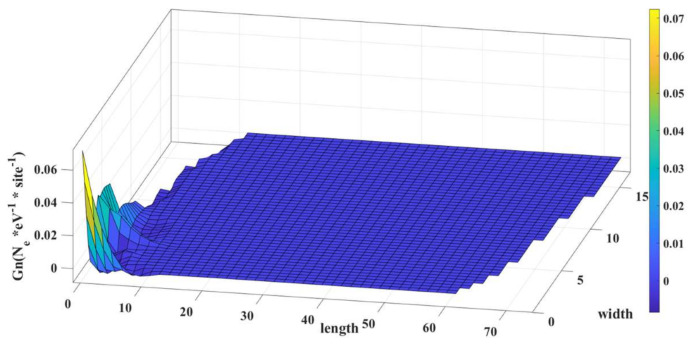
LDOS of the α-graphyne nanoribbon in OFF condition.

The bands can be vertically shifted by applying voltage to the gate. This way, we can move the Fermi level of the energy to a position in the BS where no states are available, and this will represent the off state of the device. From Figure 7, we can see the LDOS for the off condition of the device. The electron concentration along the length of the device is significantly reduced. As a result, the current conduction for the applied voltage along the length will be greatly reduced.

### 3.3. Current–Voltage Characteristics

In order to control the device’s current, we implemented the gate to create an electric field along the channel of the device. The gate is a conductive material (metal), and it is separated from the channel region by a thin layer of dielectric insulating material. The thickness of the gate oxide is 10 Å. Apart from the insulating layer and the gate, all other parts are constructed of the α-graphyne nanoribbon.

When a voltage is applied to the gate terminal, it creates an electric field along the channel, as discussed earlier, that penetrates the gate oxide and interacts with the charge carriers in the channel material, either attracting or repelling them from the gate.

**Figure 8:**
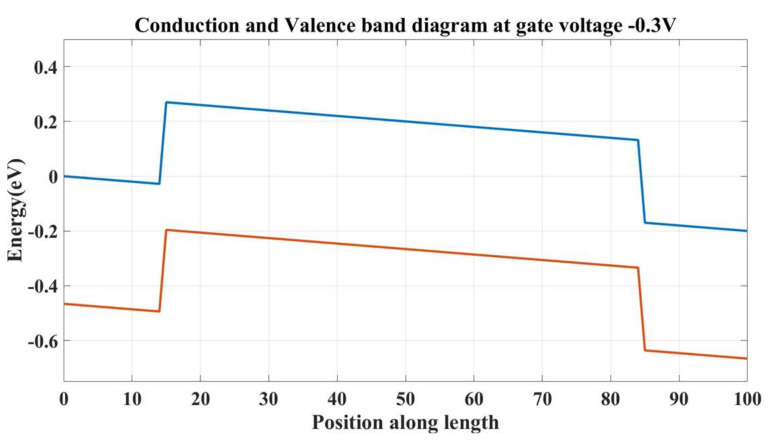
Potential profile in the off state.

When there is no gate voltage, the electrons flow from the source to the drain. When a negative voltage is applied, the energy level of the channel region rises, while the energy level of the leads remains unchanged. This results in a potential barrier that prevents the electrons from flowing through the device. This is shown in Figure 8.

Our device’s performance is highly dependent on the channel’s material. Here, silicon dioxide was selected as the insulating material, and its dielectric constant was fixed (3.9), which keeps the electric field strength at the maximum for a given input voltage. In order to achieve optimum performance, we used silicon dioxide as the insulating material.

**Figure 9:**
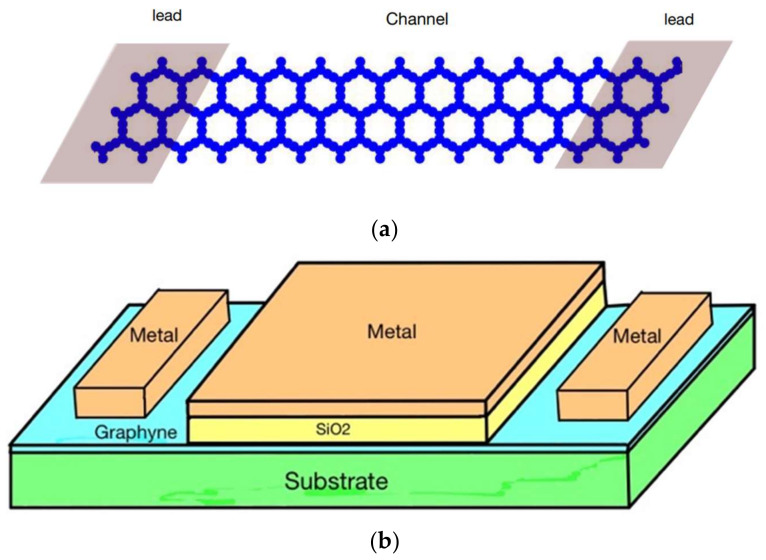
(**a**) Device structure and (**b**) complete device schematic.

For controlling the device’s current effectively, the gate length should be greater than or equal to the channel length. In our constructed device, the whole device acts as a channel region. Therefore, the gate is applied across the entire device. Also, the same material (alpha-graphyne) was used as the left and right leads to act as the source and drain terminal for the device. The structure of the device is shown in Figure 9a and the complete device schematic is shown in Figure 9b.

In our study, the Fermi level lies at Ef = 0.233 eV: applied drain-to-source voltage V_ds_ = 0.2 V. The applied gate bias varies from −0.4 V to 0.1 V.

**Figure 10:**
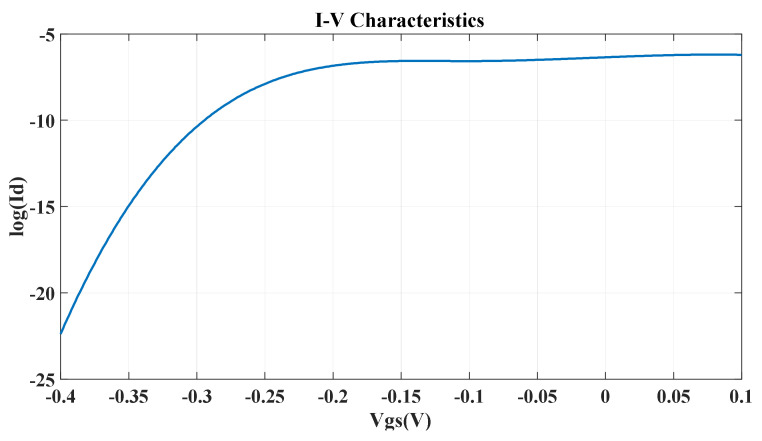
I-V characteristics of the α-GyNR-FET.

The current voltage characteristics (output current vs. gate voltage) are shown in Figure 10.

We can control the current through the device by varying the gate voltage. When we apply positive or zero voltage at the gate terminal, the overall band structure shifts downward, and we remain in a region where there are allowed energy states for the current to flow. In this region, the device is on, and a significant amount of current flows through the device. At V_gs_ = 0 V, the value of the drain current is $4.41\times 10^{-7}$ A, which is the device’s on current, i.e., Ion = $4.41\times 10^{-7}$ A.

When we apply a negative voltage at the gate terminal, the overall band structure is lifted up, and when the Fermi level falls in the bandgap, very little current will flow. In this region, the device is in the off mode. When we start to apply a negative voltage at the gate terminal, the current decreases gradually. At gate voltage V_gs_ = −0.3 V, a very small amount of drain current flows. This is the off current of the device, i.e., Ioff = $4.31\times 10^{-11}$ A. Ideally, no current should flow in the off mode. But we acquire a finite off current because of the tunneling effect. We also use this region of the on-to-off current for calculation of the subthreshold swing.

The subthreshold swing depends on the on current and the off current of the device as well as the voltage differences of the operating region [17]. The subthreshold swing is an important parameter of transistor modeling. It refers to a given voltage range and how fast the device switching mechanism can occur. Therefore, a higher value for the subthreshold swing means a larger voltage swing required for the switching mechanism of the device. As a result, a lower value for the subthreshold swing is required. The lower the subthreshold swing, the better the controllability of the device channel, which means improved Ion/Ioff [18]. For our device, the subthreshold swing is as below:

$$\begin{array}{cc} \mathrm{Subthreshold}\;\mathrm{swing} & =\frac{V_{{\mathrm{gs}}_{\mathrm{on}}}-V_{{\mathrm{gs}}_{\mathrm{off}}}}{\log10\left(I_{\mathrm{on}}\right)-\log10(I_{\mathrm{off}})}\times 1000\;\left(\frac{\mathrm{mV}}{\mathrm{dec}}\right)\\ & =\;74.8\;\mathrm{mV}/\mathrm{decade} \end{array}$$

Here, V_gson_ = 0 V and V_gsoff_ = −0.3 V and Ion = $4.41\times 10^{-7}$ A and Ioff = $4.31\times 10^{-11}$ A.

**Figure 11:**
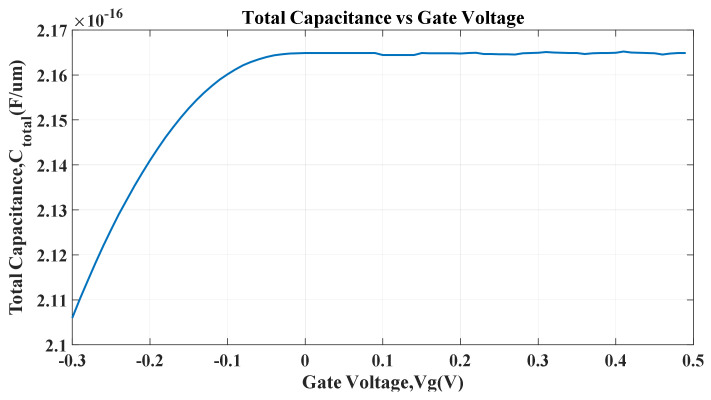
Total capacitance of the device with respect to the gate voltage.

The capacitance of the device is plotted in Figure 11.

The oxide capacitance is a constant value $7.2162\times 10^{-17}$ F/um. The oxide capacitance is calculated for a thickness of 10Å and the per unit length of the channel. In the region Vg < 0.1 V, the quantum capacitance dominates. When Vg is increased to higher values, the effect of quantum capacitance becomes negligible, and the oxide capacitance dominates the total capacitance value. Oxide capacitance and quantum capacitance constitute the total capacitance of our device.

The delay time depends on the RC constant value. The higher the RC constant value, the higher the delay time. Therefore, if the total capacitance of the device is reduced, the delay time becomes lower in value. From Equation (11), the DPD is proportional to the delay time, and the delay time directly depends on the total capacitance. The lower the total capacitance value means the lower the delay time and a lower DPD, which is our primary goal.

### 3.4. Effect of Vacancy Defects

Based on simulation results, the designed device works such that there is no presence of defects. But in practical cases/scenarios, defects exist in the device. Therefore, to make our device more realistic, we introduced vacancy defects to check how the performance parameters were affected.

Two phenomena become dominant in the case of the insertion of a vacancy. Firstly, the introduction of a vacancy to the device results in a reduction in the quantum transmission probability, which actually reduces the current. And so, the on–off current ratio will be higher.

**Figure 12:**
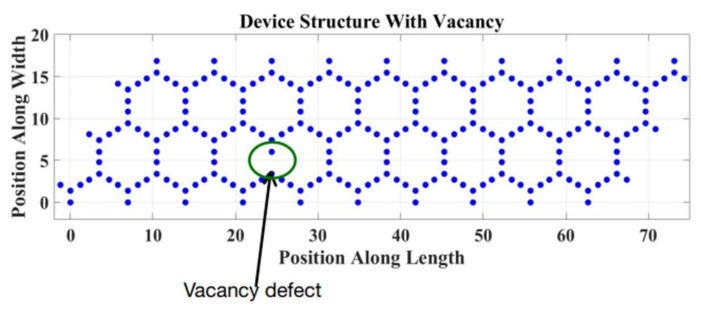
Device structure with a vacancy defect.

Secondly, the insertion of a vacancy into the device affects mostly the top layer of the electrostatic potential barrier. At the vacancy’s location, the potential barrier is increased in comparison to the ideal case, which results in smaller currents which causes the high on-to-off current ratio [18]. We introduced a single vacancy of the defect in the structure. The position of the vacancy is shown in Figure 12.

We observed the impact of the vacancy defect on the device’s performance.

In order to introduce a vacancy into the device, the Hamiltonian matrix was modified. For the particular position in the Hamiltonian matrix where we want the vacancy, the corresponding row and column intersecting the position, the zero coupling value was inserted. Therefore, no atom in the device will have any coupling with the particular position.

**Figure 13:**
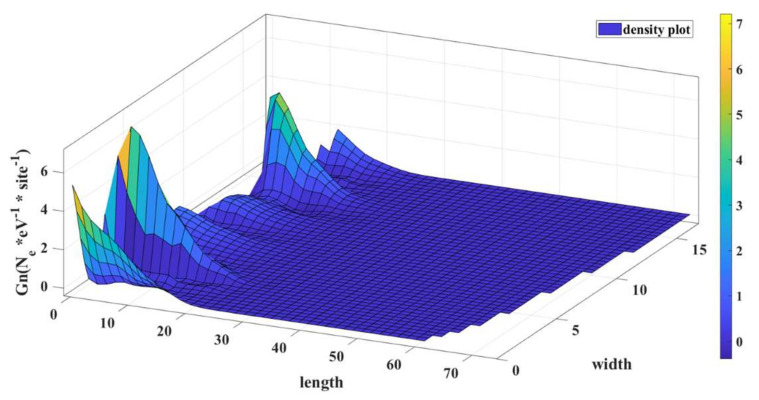
LDOS with a single vacancy defect in ON condition.

When a single vacancy defect is introduced, three nearby atoms are affected, and the electron scattering effect becomes more significant. From the LDOS profile, we can see that the electrons cannot travel after encountering the defect. This can be seen in Figure 13.

**Figure 14:**
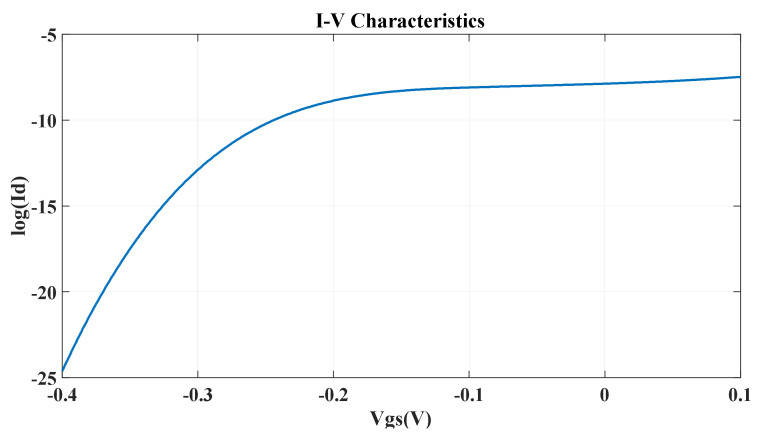
I-V characteristics for the single vacancy defect.

In Figure 14, we can see the effect of the vacancy defect on the current voltage characteristics.

We can observe the effect from the operating regions.

At Vgs = 0 V, we acquire the value of the on current Ion = $1.31\times 10^{-8}$ A.

At Vgs = −0.3 V, we acquire the value of the off current Ioff = $1.26\times 10^{-13}$ A.

The effect of the vacancy defect is more pronounced in the on current than in the off current [18]. This is because the on current is determined primarily by the source and drain contacts and their carrier injection properties. On the other hand, the off current is determined by the properties of the channel region.

The subthreshold swing in the case of the vacancy defect is calculated as:

$$\begin{array}{cc} \mathrm{Subthreshold}\;\mathrm{swing} & =\frac{V_{{\mathrm{gs}}_{\mathrm{on}}}-V_{{\mathrm{gs}}_{\mathrm{off}}}}{\log10\left(I_{\mathrm{on}}\right)-\log10(I_{\mathrm{off}})}\times 1000\;\left(\frac{\mathrm{mV}}{\mathrm{dec}}\right)\\ & =\;59.8\;\mathrm{mV}/\mathrm{dec} \end{array}$$

### 3.5. Effect of an Edge Defect

**Figure 15:**
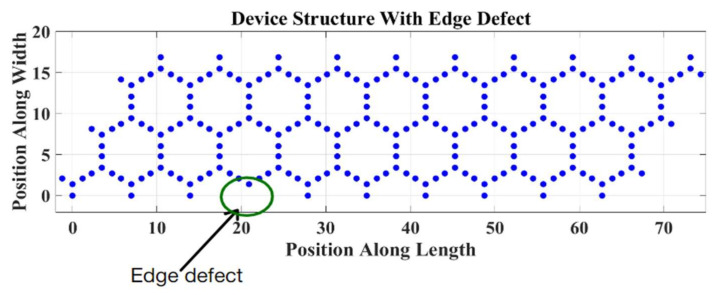
Device’s structure with an edge defect.

We added an edge defect into the device’s structure and observed its effect on the device’s performance. The position of the edge defect is shown in Figure 15.

**Figure 16:**
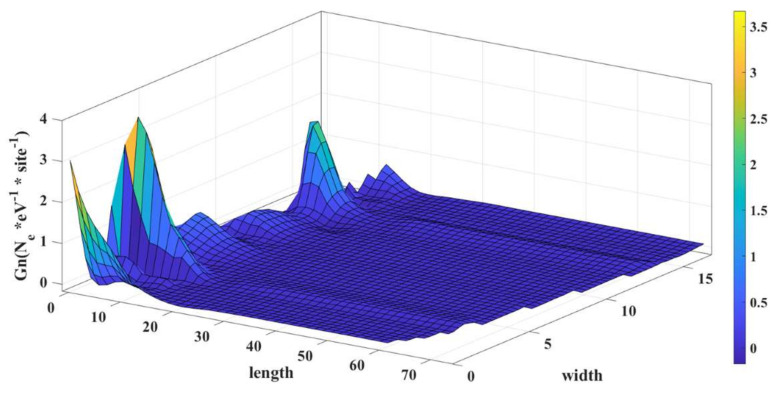
LDOS with a edge defect in the on condition.

In the case of an edge defect, only one atom is affected. The LDOS profile is shown in Figure 16.

A vacancy defect will cause a reduction in quantum transmission probability as well as reduced propagating states which will result in a smaller current [18].

**Figure 17:**
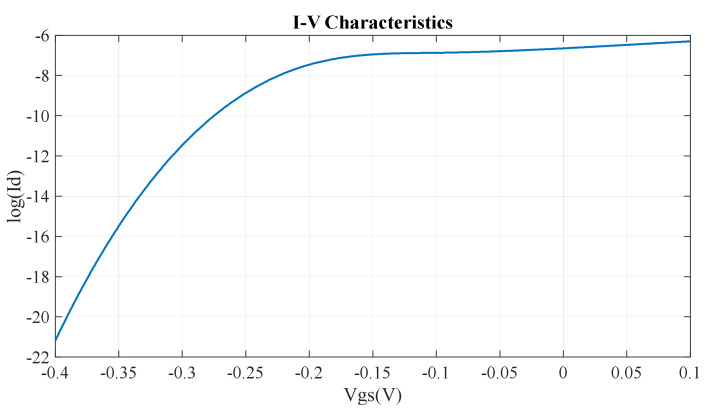
I–V characteristics for the edge defect.

The effect on the current voltage characteristics can be seen from the operating regions. This is depicted in Figure 17.

At Vgs = 0 V, we acquire the value of the on current Ion = $2.25\times 10^{-7}$ A.

At Vgs = −0.3 V, we acquire the value of the off current Ioff = $3.45\times 10^{-12}$ A.

Similar to the case of the single vacancy defect, here, we can also see that the off current is significantly affected by the edge defect, while the reduction in the on current is negligible.

The subthreshold swing in the case of the edge defect is calculated as:

$$\begin{array}{cc} \mathrm{Subthreshold}\;\mathrm{swing} & =\frac{V_{{\mathrm{gs}}_{\mathrm{on}}}-V_{{\mathrm{gs}}_{\mathrm{off}}}}{\log10\left(I_{\mathrm{on}}\right)-\log10(I_{\mathrm{off}})}\times 1000\;\left(\frac{\mathrm{mV}}{\mathrm{dec}}\right)\\ & =\;62.3\;\mathrm{mV}/\mathrm{dec} \end{array}$$

## 4. Summary of the Results

We can summarize the results in Table 1 by comparing the performance parameters for the three cases as discussed in the previous section.

From the table, there is a trade-off between the three parameters such as the subthreshold swing, the delay time, and the DPD.

In case of the lower delay time and DPD, the subthreshold swing becomes larger, which shows that the switching mechanism takes a longer time.

In case of the higher delay time and DPD, the subthreshold swing becomes smaller, which shows that the switching mechanism takes a shorter amount of time, but a higher value for the delay time and DPD can be limitations for high-speed/high-performance devices.

We can see from the table that the subthreshold swing decreases as we add defects. This can be explained as, since the off current is significantly reduced while the on current does not change much, the Ion/Ioff ratio becomes higher, reducing the subthreshold swing.

As defects are added to the device, the electrons experience backscattering. As a result, the mobility decreases and, consequently, the capacitance increases. This leads to an increase in the delay time and dynamic power dissipation (DPD).

The single vacancy defect affects the performance more than the edge defect as three neighboring atoms are affected compared with one atom in the case of the edge defect.

From Table 2, we observe that our designed subthreshold swing value is larger than the IRDS 2022 value, but the total capacitance is lower than the IRDS 2022 which signifies that the delay or response time and dynamic power dissipation (DPD) will be much lower for our designed case.

## 5. Conclusions

In this paper, we have modeled an α-GyNR FET device and observed its performance parameters both with and without adding defects. We achieved a subthreshold swing of 73.89 mV/decade without any defects. The IRDS 2022 requirement is 67 mV/decade. The on current is 4.41 × 10^−7^ A, and the IRDS 2022 requirement is 5.96 × 10^−6^ A.

The required capacitance is 0.39 fF/μm, as per the IRDS 2022, and we obtained the capacitance value of 0.2166 fF/μm, which is better than the required value. The dynamic power dissipation is 0.00864 fJ/μm, which is much lower than the IRDS 2020 requirement of 0.33 fJ/μm.

After adding a single vacancy defect, we achieved a subthreshold swing of 59.8 mV/decade, and with an edge defect, we achieved a value of 63.29 mV/decade, which is lower than the IRDS 2022 requirement of 67 mV/decade. But the trade-off is that the power dissipation and delay time increases after the defects are introduced. Our primary goal is achieving a lower subthreshold swing, a low delay time, and a low DPD from our designed FET device, and we achieved satisfactory results for the parameters mentioned. Moreover, for this particular dimension (a width of 1.69 nm and a length of 7.56 nm), it is possible to fabricate the FET device at the hardware level. Therefore, it is possible to design a FET device that meets the performance requirements using alpha-graphyne.

## Figures and Tables

**Table 1:** Comparison between the without defect, vacancy defect, and edge defect scenarios.

	Without Defect	Vacancy Defect	Edge Defect	
Parameter	Value	Value	Value	Unit
Ion	4.41 × 10^−7^	1.31 × 10^−8^	2.25 × 10^−7^	A
Ioff	4.31 × 10^−11^	1.26 × 10^−13^	3.45 × 10^−12^	A
Ion/Ioff	10,248	103,770	65,195	
Subthreshold Swing	74.8	59.8	62.3	mV/decade
$\tau_{d}$	0.7413	24.9	1.45	ps
DPD	0.008664	0.00867	0.008677	fJ/μm
Total Capacitance	0.2166 × 10^−15^	0.2167 × 10^−15^	0.2169 × 10^−15^	F/μm

**Table 2:** Comparison of the device parameters (without defects) with the IRDS 2022.

	Designed Case	IRDS 2022	
Parameter	Value	Value	Unit
Ion	4.41 × 10^−7^	5.96 × 10^−6^	A
Subthreshold Swing	73.89	67	mV/decade
Total Capacitance	0.2166	0.39	fF/μm

## Data Availability

Not applicable.
